# Study on the rate of plutonium vertical migration in various soil types of Lublin region (Eastern Poland)

**DOI:** 10.1007/s10967-013-2774-6

**Published:** 2013-10-11

**Authors:** Jolanta Orzeł, Andrzej Komosa

**Affiliations:** Department of Radiochemistry and Colloid Chemistry, Maria Curie-Sklodowska University, Lublin, Poland

**Keywords:** Plutonium isotopes, Alpha spectrometry, Migration rate, Soil profile

## Abstract

Soil contamination level with ^239+240^Pu of Lublin region was determined using the alpha spectrometric method. Results were compared with similar data from the study performed 15 year earlier. Decrease in total ^239+240^Pu concentration and reducing quantity of Chernobyl fraction (up to almost negligible value of 1 %) has been observed in upper soil layer. Determination of ^239+240^Pu concentration in soil profile layers allows calculating a vertical migration velocity of plutonium applying a compartment migration model. It was found that ^239+240^Pu migration rate varies depending on soil type from 0.29 cm year^−1^ in Podsols to 0.58 cm year^−1^ in Fluvisols with mean value of 0.5 cm year^−1.^

## Introduction

Alpha radiating plutonium isotopes are still present in the environment, especially in upper layers of soil. Despite of many years passed since the events which caused introducing rather large plutonium amounts into the environment (the global fallout from the atmospheric nuclear weapon tests carried out between 1945 and 1980 and the Chernobyl disaster in 1986) and its global dispersion, the current sensitive spectrometric methods allow still detecting this element in the environment [1]. It especially concerns soils because of the fact that the fallout plutonium was accumulated in soil surface layer and is a subject of various physicochemical processes leading to its movement downward the soil profile [2].

Currently, all amount of plutonium nuclides can be found on the Earth surface bound with soil components, penetrating down the soil profiles, being dispersed in the ground-level air (as a result of re-suspension) or finally passing into sediments. Movement rate of radionuclides in the environment is influenced by many factors as radionuclide chemical form, chemical composition of soil, physicochemical conditions, quantity of atmospheric precipitation, presence of vegetation and its kind, the micro-organisms and small animals activities, and human activity as well. Additionally, transuranic elements reveal rather high ability to form radio-colloids what enhances its environmental mobility [3, 4].

Plutonium studies, apart from purposes connected with determination of plutonium alone, give a possibility to characterize the behavior of other, non-radioactive trace elements at concentrations below a limit of detection of various analytical methods. Radionuclide can be treated as a tracer to display, for example, relocation of heavy metals. Determination of plutonium by alpha spectrometry requires a great deal of work, especially during radiochemical treatment of the sample. This is necessary for preparation of a pure plutonium sample suitable for the alpha spectrometric measurement.

Plutonium isotopes make a potential hazard for human life because of emission of the alpha radiation with high ionizing power. If plutonium gets inside the human body, mainly as a result of inhalation of re-suspended soil particles, it can impact a man health. Recently, an increasing interest of the environment contamination status has been observed in Poland. It concerns a current level of natural and anthropogenic radionuclides in the environment. This interest is a result of planning to build in Poland the first nuclear power plant. In the case of an emergency release of plutonium (generated during a nuclear fuel burn-up process) a long-lasting contamination could arise.

Studies on determination of radioisotope concentration level in soils of Lublin region were started in 1996 [5] and continued in 1999 [6]. Particularly, a vertical transport of different radionuclides in soil [6, 7], their binding with the soil fractions with different physicochemical properties using sequential extraction [8], transfer to plants [9] and animals [10] has been studied.

Numerous papers on the vertical distribution of radionuclides in soils, including plutonium, have been published. Usually, the activity-depth profiles are modeled using a suitable set of equations based on adequate assumptions. In the former papers a simple exponential model was used (e.g. [11, 12]). Later, a compartment model based on residence time of radionuclide in different soil layers [12–15] and the advection–dispersion models have been developed simultaneously [12, 13, 15–17]. The compartment model is rather simple and does not require as many known or assumed parameters as the others do. However, the advection–dispersion models are still improved to better fit measurement data. Recently, bioturbation processes occurring in soil (causing by burrowing animals as pocket gophers, earthworms or ants) have been included in the model [18, 19].

The aim of this study was the determination of plutonium contamination level of various types of soils on the area of Lublin region. Calculation of the vertical plutonium transport velocity downward the soil profile was also performed. The results were compared with older ones to find the changes of plutonium activities in soil profiles during the last 15 years.

## Materials and methods

Lublin region is located on the area of several physico-geographical regions, different in respect of relief, landscape and vegetation.

**Fig. 1:**
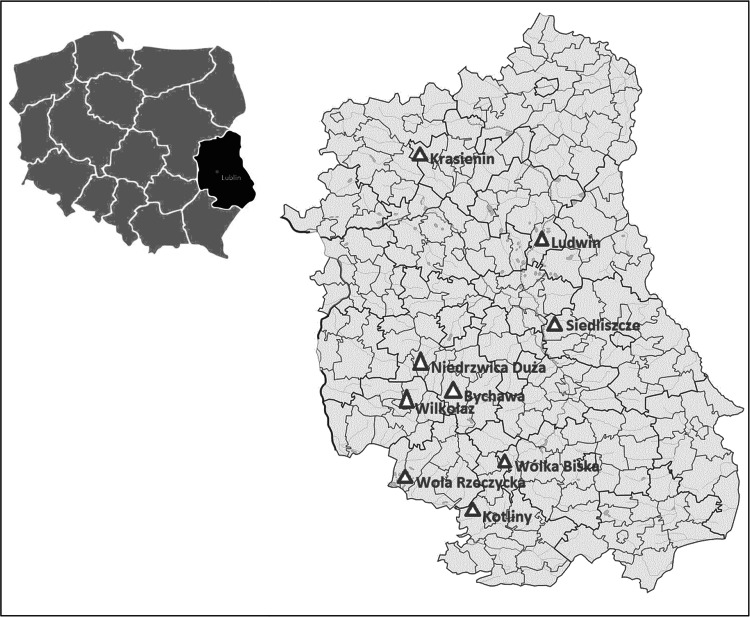
Localization of sampling points

Soil profile samples were collected on the area of the Lublin region, as presented in Fig. 1, where brown earths, build on loess formations, are a predominating soil types. Samples of soil profiles were collected from uncultivated area from spring to autumn in a period 2008–2010 by means of cylindrical cores (10 cm diameter). In every place three cores were taken (at a distance of one meter between them) which afterwards were open in the laboratory to expose the profile horizons. After identification of the soil type and a profile structure the core was divided into 5 cm layers. The respective layers of three cores were combined to form the sample submitted to plutonium determination. Characteristics of samples are present in Table 1, where organic matter contents (OM %) calculated as a loss during ashing and exchangeable pH (measured in 1 M KCl solution) are presented. These values are arithmetic mean from those obtained for each layer separately.

**Table 1:** Sample collection places and soil characteristics

Locality (acronym)	Geographical coordinates	Soil type	Mean OM content (%)	Mean exchangeable pH
Wilkołaz (WIL)	N-51^°^01′08.46″	Fluvisols	42.3	5.35
	E-22^°^21′14.84″	Chernozems		
	Height 227 MASL			
Bychawa (BYCH)	N-51^°^02′23.90″	Fluvisols	4.98	6.49
	E-22^°^53′53.70″			
	Height 222 MASL			
Krasienin (KRA)	N-51^°^21′41.60″	Fluvisols	6.05	5.73
	E-22^°^27′41.30^″^			
	Height 194 MASL			
Niedrzwica uża (NIE)	N-51^°^07′12.78″	Histosols	0.72	3.99
	E-22^°^23′03.13″	Chernozems		
	Height 194 MASL			
Ludwin (LUD)	N-51^°^21′05.16″	Histosols	8.81	7.39
	E-22^°^54′35.40″	Chernozems		
	Height 165 MASL			
Wólka Biska (WOL)	N-50^°^26′56.43″	Histosols	2.95	5.11
	E-22^°^36′18.07″	Chernozems		
	Height 174 MASL			
Siedliszcze (SIE)	N-51^°o^11′44.21″	Histosols	9.71	7.35
	E-23^°^9′42.43″	Muck soil		
	Height 180 MASL			
Wola Rzeczycka (WRZ)	N-50^°^39′30.80″	Gleysols	9.18	3.81
	E-22^°^0′53.90″			
	Height 144 MASL			
Kotliny (KOT)	N-51^°^29′07.28″	Podsols	6.26	5.54
	E-22^°^09′07.64″			
	Height 173 MASL			

**Fig. 2:**
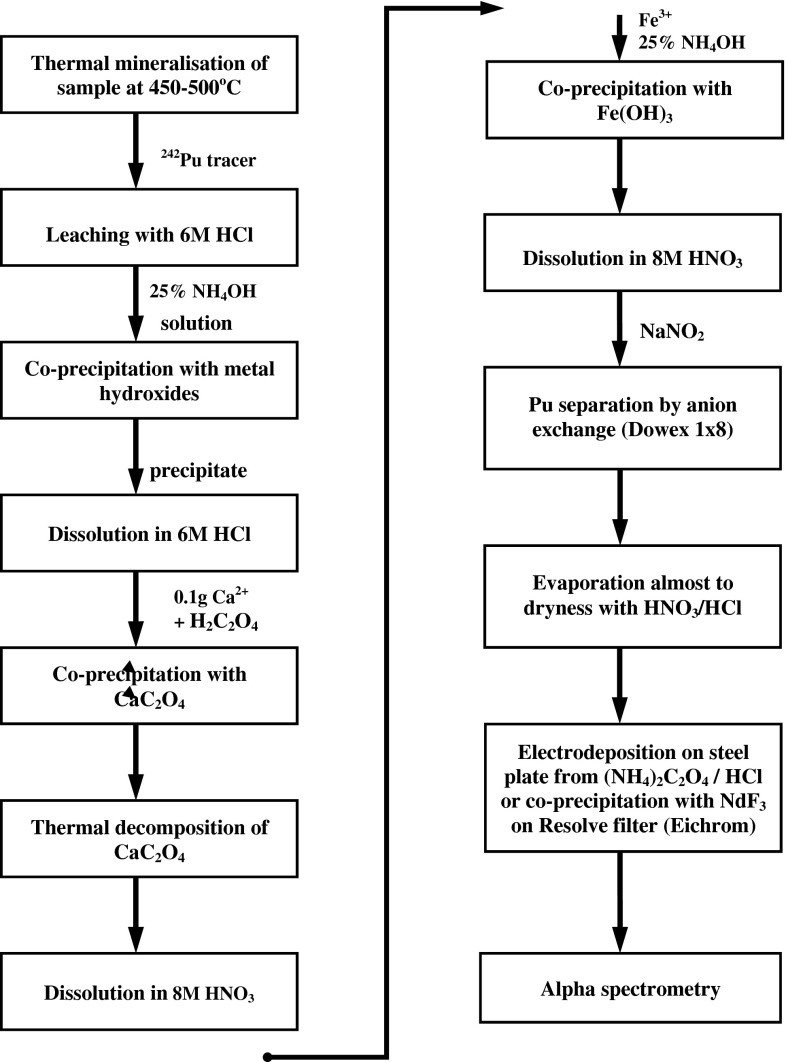
Scheme of radiochemical procedure for Pu separation

Plutonium isotopes were determined in sub-samples (50 g dry mass) taken from every soil layer of collected profiles. Applied radiochemical procedure for plutonium separation and measurement by alpha spectrometry (based on IAEA guidebook [20]) is presented in Fig. 2. The standard solution of ^242^Pu of activity concentration 0.73 Bq g^−1^ (AEG Fuel Services, UK) as a yield monitor was used. Sample of chemical recovery less than 50 % was rejected and Pu determination was repeated with a new sub-sample. All measured activities exceeded the value of minimum detectable amount which amounted from several mBq kg^−1^ up to 25 mBq kg^−1^. Alpha activity measurements were performed using four alpha spectrometers (Canberra, Model 7401), connected with a mixer-router 1520 and S-100 multichannel analyzer (Canberra). The PIPS detectors of 17 keV FWHM resolution were used and the Genie 2000 (version. 2010) software for quantitative analysis applied. Uncertainty of Pu determination varied from 5 to 15 % and is presented in Tables 3, 4, 5, and 6 together with each result. To validate our procedure the standard reference materials have been analyzed (IAEA 384 and IAEA Soil-6). The result of ^239+240^Pu determination of IAEA 384 sample was 111 ± 13 Bq kg^−1^ (certified value 108 Bq kg^−1^) and IAEA Soil-6: 1.065 ± 0.131 Bq kg^−1^ (certified value 1.036 Bq kg^−1^). Our laboratory passed also the IAEA Proficiency Test in 2013 on determination of ^239+240^Pu in soil.

Knowledge of plutonium concentration in particular layer of soil profiles serves to calculate a vertical transport rate down the soil profile. Compartment model was used for this purpose. This was chosen because of small number of parameters needed to obtain the velocity results. The model assumes that plutonium migrates downward the profile from the one (upper) do the second (lower) compartment (soil layer) and the concentration inside entire compartment is the same. Knowing a date of radionuclide deposition on the first compartment and the time period from the deposition to measurement one can calculate a residence time of radionuclide in every compartment and a velocity of migration from one compartment to another [12, 16, 17].

## Results and discussion

**Fig. 3:**
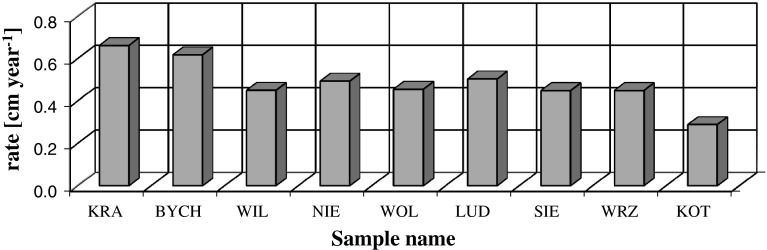
Mean velocity of ^239+240^Pu migration in the 0–15 cm layer (cm year^−1^)

**Fig. 4:**
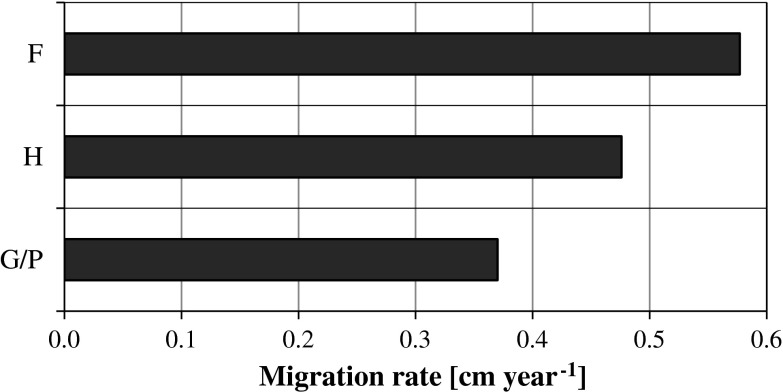
Mean velocity of ^239+240^Pu migration in the 0–15 cm layer (cm year^−1^) for three types of soil: F Fluvisols, H Histosols, G/P Gleysols/Podsols

Results of the study are presented in Tables 2, 3, 4, 5 and 6 and in Figs. 3 and 4. Table 2 presents a total contents of ^239+240^Pu in particular soil profile expressed as plutonium fallout (in Bq m^−2^) together with a fraction of ^239+240^Pu of Chernobyl origin which was calculated taking into account an isotopic ratio ^238^Pu/^239+240^Pu [5] after decay correction to the date of measurement.

**Table 2:** Total fallout of ^239+240^Pu (Bq m^−2^) and its mean percentage of Chernobyl origin in total profile

Profile name	Total fallout ^239+240^Pu (Bq m^−2^)	Chernobyl ^239+240^Pu fraction (%)
WIL	69.0	1.2
BYCH	60.3	1.4
KRA	82.4	0.2
NIE	29.9	1.2
LUD	60.9	1.7
WOL	34.3	0
SIE	51.0	0
WRZ	45.8	0.6
KOT	50.0	2.1
Mean	54 ± 26	0.9 ± 1.1
Global fallout in Poland (UNSCEAR1982)	58

**Table 3:** The ^239+240^Pu concentration in soil profile layers of Fluvisols

Layer (cm)	KRA	BYCH	WIL
	^239+240^Pu (mBq kg^−1^)		
0–5	97 ± 8	133 ± 8	199 ± 10
5–10	89 ± 6	119 ± 7	402 ± 26
10–15	122 ± 9	142 ± 8	387 ± 18
15–20	203 ± 18	133 ± 8	306 ± 15
20–25	202 ± 11	156 ± 9	183 ± 9
25–30	79 ± 6	109 ± 7	151 ± 19
30–35	106 ± 8	74 ± 7	88 ± 7
35–40	49 ± 6	34 ± 5	93 ± 8

**Table 4:** The ^239+240^Pu concentration in soil profile layers of Histosols

Layer (cm)	NIE	WOL	LUD	SIE
	^239+240^Pu (mBq kg^−1^)			
0–5	151 ± 10	98 ± 5	143 ± 8	109 ± 5
5–10	107 ± 11	138 ± 11	190 ± 9	126 ± 8
10–15	39 ± 4	51 ± 4	227 ± 13	168 ± 12
15–20	42 ± 3	45 ± 4	225 ± 14	122 ± 9
20–25	73 ± 8	29 ± 3	128 ± 10	97 ± 9
25–30	17 ± 2	37 ± 4	157 ± 11	27 ± 4
30–35	12 ± 2	24 ± 3	194 ± 10	93 ± 9
35–40	8 ± 2	36 ± 6	48 ± 4	16 ± 2

**Table 5:** The ^239+240^Pu concentration in soil profile layers of Gleysols (WRZ) and Podsols (KOT)

Layer (cm)	WRZ	KOT
	^239+240^Pu (mBq kg^−1^)	
0–5	209 ± 12	150 ± 11
5–10	214 ± 2	168 ± 12
10–15	202 ± 11	153 ± 9
15–20	216 ± 13	134 ± 8
20–25	78 ± 1	35 ± 4
25–30	58 ± 0.9	18 ± 3
30–35	44 ± 2	5 ± 2
35–40	45 ± 1

**Table 6:** Mean residence time of ^239+240^Pu in soil layers (year) and its vertical migration rate (cm year^−1^)

Profile name	Mean residence time (year)	Vertical migration rate (cm year^−1^)
WIL	11 ± 1.7	0.45 ± 0.07
BYCH	9.1 ± 4	0.62 ± 0.26
KRA	8 ± 2.5	0.66 ± 0.18
NIE	15 ± 9.4	0.49 ± 0.43
LUD	10 ± 2.4	0.5 ± 0.1
WOL	13 ± 5.7	0.46 ± 0.26
SIE	11 ± 2	0.45 ± 0.08
WRZ	11 ± 2.5	0.45 ± 0.11
KOT	18 ± 4	0.29 ± 0.06

The data presented in Table 2 show that plutonium fallout on the area of Lublin region only slightly differ from the literature value found for the latitude 40–50°N [21]. This indicates the global fallout as a source of plutonium in Lublin region. This is also confirmed by a value of calculated Chernobyl fraction of ^239+240^Pu being at the level of 1 % only. Comparison of obtained values of total plutonium fallout with previous measurements from 1999 (sample collected in 1996) [6] does not show any important differences: presented in this paper (Table 2) mean value of plutonium fallout amounted 54 ± 26 (range 30–82) Bq m^−2^ and previous value [6] was 51 ± 10 (range 40–60) Bq m^−2^. However, the Chernobyl fraction of ^239+240^Pu in 1999 ranged from 0 to 20 % in particular layers being an average 3.6 and 9.5 % in two forest soil profiles [6].

The current results were also compared with older literature data from the year 1996 (samples collected in 1993–1994) [5]. Because the results in mentioned paper [5] were presented as plutonium concentration in an upper soil layer, expressed in Bq kg^−1^, a comparison was performed basing on the concentrations in the first layer of soil profiles (see Tables 3, 4 and 5). The results of mean plutonium concentration in present study and previous one [5] were: 143 ± 56 and 208 ± 85 Bq kg^−1^, respectively. This difference is important and arises not only as a result of higher Chernobyl fraction (which was at a level of 1 % in the present study and 14 % in the previous one). Considering only the global fallout fraction, the older data [5] still reveal higher plutonium concentration (about 1.26-times). Undoubtedly, this is an evidence of vertical plutonium migration during the last 15 years. Decrease of the Chernobyl fraction in surface soil in that period confirms our earlier observation on faster migration of Chernobyl fraction of plutonium [6].

Results of ^239+240^Pu concentration in particlular layers of soil profiles are grouped by similar type of soil: Fluvisols (Table 3), Histosols (Table 4) and Gleysols and Podsols (Table 5).

As can be seen from Tables 3, 4 and 5, maximum concentration of plutonium appears in a few upper layers, usually 0–20 cm depth. In two samples of Fluvisols (KRA and BYCH) the maximum concentration is observed deeper, in the 10–25 cm depth layer. On the other hand, in two samples of Histosols (NIE and WOL) maximum ^239+240^Pu activity concentration is seen in the 0–10 cm layer. These observations can not be simply related with organic matter contents nor with exchangeable pH measured for each layer of soil profile. There is no definite correlation between these parameters of soil and plutonium concentration.

Basing on the results presented in Tables 3, 4 and 5 the vertical migration velocity of ^239+240^Pu downwards the profile was determined by using the compartment model, mentioned above. For simplification, it was assumed that plutonium fallout took place once in 1963 where maximum of atmospheric concentration of plutonium occurred. The data presented in Table 2 clearly suggest that the fraction of Chernobyl ^239+240^Pu can be neglected. The results of calculations can be seen in Table 6 and Figs. 3 and 4.

Using the compartment model the values of residence time of plutonium in particular layers (compartments) was calculated as well as a migration rate from upper to lower layer. In deeper soil layers the plutonium concentration is small, therefore is determined with higher uncertainty. That is why, a value of calculated migration rate is incredible. For this reason values shown in Table 6 and in Figs. 3 and 4 were limited to the depth range 0–15 cm.

Table 6 presents the mean residence time of ^239+240^Pu in each of 5-cm layers (in the first three layers of the profile) and the calculated mean vertical transport rate of plutonium between soil layers. It is seen that residence time ranges from 8 to 18 years depending on the depth, and average migration rate amounts 0.48 ± 0.11 cm year^−1^. Values of plutonium migration velocity is presented in Fig. 3 for all samples.

Considering the type of soil one can see that some relation appears between value of migration rate and type of soil. This dependence is shown on Fig. 4. As it is seen, ^239+240^Pu from global fallout moves the most rapidly in Fluvisols (WIL, BYCH, KRA) amounting to 0.58 ± 0.11 cm year^−1^, medium fast in the case of Histosols (KRA, NIE, LUD, WOL and SIE): 0.47 ± 0.02 cm year^−1^, and slowly in Podsols (KOT): 0.37 ± 0.11 cm year^−1^. These differences in migration velocity can not be connected with such soil parameters as organic matter contents or exchangeable pH, as no correlation were found in these cases.

## Conclusions

The study was concerned firstly an assessment of contamination level of soil from Lublin region with plutonium isotopes and secondly, calculation of vertical migration rate of plutonium downward the soil profile of various type of soils. It was found that the total ^239+240^Pu concentration in the profiles corresponds to value of a global fallout contamination. This was additionally confirmed by the negligible ^239+240^Pu Chernobyl fraction (about 1 %) presence. Comparing the results with other ones, published 15 years earlier, proves the diminishing of ^239+240^Pu level in the surface soil and considerable reduction of the Chernobyl plutonium fraction, what confirms its quicker migration. Measured ^239+240^Pu concentration in soil layers reached a value of 400 mBq kg^−1^.

The compartment model allowed to calculate a velocity of plutonium migration, which amounted on average 0.5 cm year^−1^. At the same time the differences in migration rate were observed depending on type of soil. However, no correlation was found between plutonium velocity and such soil parameters as exchangeable pH and organic matter contents.
